# The prevalence and long-term effects of PTSD and moral injury in Swedish military veterans

**DOI:** 10.3389/fsoc.2025.1499411

**Published:** 2025-05-26

**Authors:** Sofia Nilsson, Alicia Ohlsson, Sofia Svensén, Gerry Larsson

**Affiliations:** ^1^Department of Leadership and Control & Command, Swedish Defence University, Karlstad, Sweden; ^2^Department of Health and Welfare, Inland University, Elverum, Norway

**Keywords:** moral injury, PTSD, mental health, military deployment, trauma

## Abstract

**Introduction:**

In the context of international military operations, officers and soldiers are exposed to various service-related stressors that may have long-lasting effects on their health and daily functioning. This study explored (1) the prevalence of symptoms indicative of both post-traumatic stress disorder (PTSD) and moral injury (MI), (2) the relationship between these conditions, and (3) the relationship between these conditions and a selection of background variables in Swedish military veterans who have previously been deployed in operations.

**Methods:**

The study was a self-report survey. Of 6000 individuals invited to participate, 1940 completed the questionnaire, resulting in a response rate of 32%. Data was analyzed using correlation and regression analyses to explore potential statistical relationships between variables of interest. Additionally, the data were also analyzed using between-group analyses (*t*-tests) to examine differences between different groups.

**Results:**

The results indicate that a low proportion of participants showed a prevalence of indications of PTSD, which are comparable to previous Swedish studies on deployed veterans. An even smaller proportion was found to show indications of moral injury when compared to the assessment of PTSD. However, besides the small group that fulfilled the cutoff score criteria, a number of respondents reported milder symptoms of both psychological and moral distress. The second goal of the study was to examine the relationship between indications of PTSD and indications of moral injury. The findings suggest that there is a considerable overlap between the two constructs. In addition, the results suggest that the risk of PTSD and MI is highest when an event is perceived as both highly stressful (fear-based) and morally challenging. The MI symptom subcluster shame accounts for the largest variance in the PTSD indicator scale within the study sample. Health- and deployment-related background variables were identified that may be related to indications of moral injury.

**Discussion:**

The study results highlight the type(s) of stressful experience and the health- and deployment-related factors that should be monitored post-deployment, which may serve as risk factors in developing indications of moral injury. The higher prevalence of indication of PTSD and MI in the past month, despite receiving various forms of support upon returning from deployment, highlights the need for MI-specific treatment.

## Introduction

In the context of international military operations, officers and soldiers are exposed to various service-related stressors, which may have long-lasting effects on their health and level of functioning in everyday life (Griffin et al., 2019; Moore et al., 2017). These stressors may include exposure to extremely stressful experiences involving actual or threatened death, serious injury or sexual violence, experienced directly, witnessed firsthand or learned to have happened to a close family member or to a close friend, the effects of which are categorized under the mental health disorder known as post-traumatic stress disorder (PTSD). PTSD includes symptoms such as reliving the event, avoiding reminders of the event, having more negative thoughts and emotions than before the event, and feeling on edge, which cause significant distress and make everyday life difficult (Blevins et al., 2015; Weathers et al., 1993).

The implications of exposure to trauma may also include other stress responses, which are less discussed in military research, such as exposure to potentially morally injurious events (PMIEs). Exposure to such events may also have long-lasting negative effects on health and wellbeing, thereby resulting in a moral injury (Litz et al., 2009; Shay, 2014). However, previously deployed Swedish military veterans go through several selection processes and represent a well-functioning societal group, and to date, research has indicated that a majority of military veterans do relatively well, mentally and physically, after returning from international deployment (Larsson et al., 2017; 2020; Nilsson et al., 2024). For example, they have had a lower incidence of mental health problems in comparison with the general population (Neovius Pousette et al., 2023b). Nevertheless, there is research indicating that a smaller group of military veterans have significant mental and physical difficulties after deployment. A recent cohort study showed a significantly higher prevalence of PTSD diagnosis from physicians in specialist care among military veterans after returning from international deployments compared to individuals who had not been deployed abroad (Neovius Pousette et al., 2023b). While PTSD is well-studied in a U.S. military context (e.g., Schein et al., 2021), less is known about the prevalence of PTSD among Swedish military veterans as a group.

In recent times, there has been a growing awareness that the emotional and psychological implications of experiencing difficult situations are complex and that some psychological impacts cannot be understood within the well-defined framework of PTSD (Blevins et al., 2015; Weathers et al., 1993). Even after PTSD-related symptoms are reduced, an internal conflict may persist, causing the individual military veteran to continue to suffer in terms of guilt and shame (Koenig and Al-Zaben, 2021). Thus, it is suggested that moral injury represents a specific type of moral trauma characterized by guilt, shame, an existential crisis, and loss of trust. This can occur when an individual has committed, failed to prevent, or witnessed actions that violate deeply held values, leading to a sense of being overwhelmed by acting contrary to one’s own moral compass in high-stakes situations (e.g., Frankfurt and Frazier, 2016; Griffin et al., 2019; Moore et al., 2017; Shay, 2002; Tick, 2005).

It has been suggested that moral injuries are often found in military personnel with PTSD because traumatic military experiences tend to create ethical dilemmas (Barnes et al., 2019). In addition, it has been proposed that moral injury occurs when the individual attempts to avoid the moral pain, ‘burden of conscience’ or shame, which is described as “an intensively painful feeling or experience of believing we are flawed and therefore unworthy of acceptance and belonging” (Brown, 2006, p. 45). This is associated with the traumatic situation and is followed by negative self-evaluation (Briere and Scott, 2006). Ultimately, this association may result in a sense of damage to one’s moral compass that changes the individual’s self-identity, reinforcing internal disengagement and impaired moral functioning (Dombo et al., 2013). There are studies of moral distress (Larsson et al., 2018; Nilsson et al., 2015, 2017) and moral injury in Swedish military contexts (e.g., Grimell, 2023; Grimell and Nilsson, 2020). However, to date, no studies have attempted to target a large-scale Swedish veteran population. Thus, little is known about the prevalence or range of the health-related effects of moral injury among Swedish military veterans as a group or of the other associated variables.

Both PTSD and moral injury are significant health outcomes because they represent long-term, chronic negative health conditions associated with serious functional impairments in important areas, such as interpersonal relationships, professional functioning, quality of life, and overall wellbeing (Griffin et al., 2019; Schein et al., 2021). While PTSD is a well-established psychiatric diagnosis with a well-defined symptom profile, moral injury is not currently considered a mental disorder (Barnes et al., 2019). Furthermore, the construct is relatively new, with no consensus definition, and is still subject to construct validation. Nevertheless, the general conception is that the two conditions are associated, potentially even overlapping, which creates methodological challenges in differentiating between the two. For that reason, scholars have explored potentially unique symptom profiles and mediators that may separate the two trauma types (Griffin et al., 2019). In their integrative review, Griffin et al. (2019) have found that PTSD and moral injury are associated but distinct, separate responses to trauma exposure. For example, the former is fear-based, while the latter includes core indications related to self-blame, trust, and spiritual/existential issues. PTSD includes “exaggerated startle reflex, memory loss, flashbacks, nightmares, and insomnia while the moral injury profile comprises guilt, shame, anger, anhedonia, and social alienation” (Griffin et al., 2019, p. 352).

It may also be noted that perpetration-based events have been shown to be more closely associated with symptoms such as reexperiencing guilt and self-blame when compared with life-threatening traumas (Litz et al., 2018). Thus, Griffin et al. (2019) conclude that moral injury-related health outcomes cannot be reduced to PTSD symptoms alone. Some scholars compare moral injury with symptoms experienced in complex PTSD (CPTSD), as the latter includes both PTSD symptoms and additional symptoms such as feelings of worthlessness, shame, and guilt from experiencing recurring traumas or a long-term traumatic event (Currier et al., 2021).

In a military context, it is important to consider that life-threatening traumatic and potentially morally injurious events (PMIEs) take place within the individual’s professional practice. For example, these PMIEs can include seeing injured women and children and not always being able to help, finding oneself in a life-threatening situation and not being able to respond due to rules of engagement, or experiencing leader betrayal in high-stakes situations (Hansen et al., 2021). Thus, the individual’s options for action are usually constrained by external factors. Therefore, it is proposed that both PTSD and moral injury present challenges at the societal level because they connect personal difficulties related to the generally accepted social processes of the military organization. For example, such challenges could include following a superior’s orders, which you disapprove of, or having to kill another human being. Therefore, the prevailing tendency to prioritize clinical treatment after trauma at the expense of preventative strategies poses significant challenges. In addition, the relatively unilateral focus on PTSD-related symptoms in military contexts tends to neglect the potential occurrence of other stress responses, such as moral injury (Steenkamp et al., 2015).

Based on the discussion above, this study intends to explore (1) the prevalence of PTSD and moral injury indications, (2) how these conditions relate to each other, and (3) how they are related to a selection of background variables, in Swedish military veterans who have previously been deployed. An additional ambition of this study is to contribute to bridging the gap between theory and empirical investigation to create a greater understanding of Swedish military veterans’ perceived experience of both constructs.

## Methods

### Participants and procedure

The study is based on data from military veterans who have been deployed abroad. With the support of the Veterans Center in the Swedish Armed Forces (SAF), 6,000 individuals were invited to participate in the study. They were randomly selected based on the three strata from the Swedish Veteran registry below:

- 2,000 individuals who served abroad approximately 0–10 years ago (Mali, Afghanistan, Tchad),
- 2,000 individuals who served abroad approximately 11–20 years ago (Kosovo, Afghanistan), and
- 2,000 individuals who served abroad approximately 21–33 years ago (Bosnia, Kosovo).

The three strata roughly correspond to the Swedish Armed Forces’ international peace enforcement deployments (United Nations Charter, Chapter VII) in Mali, Afghanistan, and Bosnia.

The potential respondents were contacted via a mail during the spring of 2024 to inquire about their willingness to participate in the study. The mailing included a paper questionnaire with a prepaid return envelope, a link to a digital version of the questionnaire, an information letter prepared by the project team with information about the specific project, and an information letter from the SAF Veterans Center about available support resources. The questionnaire was completed by 1,940 individuals, with a response rate of 32%. Table 1 gives an overview of the background characteristics of the respondents.

**Table 1:** Background characteristics of the military veteran sample.

Military veteran sample *N* = 1929	*n*	(%)
Variable		
Sex		
Women	169	9
Men	1758	91
Age		
<31	65	3.4
31–40	304	15.9
41–50	497	26
51–60	665	34.8
61–70	251	13.1
>70	129	6.8
Civil education		
Elementary school	47	2.5
Secondary education	668	34.5
University education – not graduated	303	15.5
University education – graduated	912	47.5
Marital status		
Married/common-law	1,488	77
Single	442	23
Employment in the armed forces		
Yes, permanent	391	20
Yes, intermittent	293	15
Yes, civilian	112	6
No, have other employment	869	45
No, unemployed	13	1
No, study	18	1
No, retired	230	12
Total length of employment in the Armed Forces		
<1 year	274	14
1–3 years	527	27
4–10 years	364	19
>10 years	763	40
Military branch		
Army	1,400	73
Navy	174	9
Air Force	206	11
Other	135	7 (multiple)

The majority of the study participants were men, aged between 41 and 60 years, and held university degrees. Many had also graduated from secondary education. The vast majority were married or cohabiting. While many were still employed in the armed forces (either permanently, intermittently, or as civilians), a significant portion also had civilian employment. The majority had served for 10 years or longer and had an army background.

Table 2 provides an overview of health-related background characteristics.

**Table 2:** Health-related characteristics of the military veteran sample.

Military veteran sample	*n*	(%)
Variable		
Previous trauma	428	23
Yes	1,429	77
No		
Previously diagnosed with PTSD		
Yes	69	3.7
No	1805	95.7
Prefer not to answer	12	0.6
If yes, when?		
Before the deployment accounted for	3	2.9
After the deployment accounted for	64	61.5
Prefer not to answer	37	35.6
Sufficient support/recovery after the deployment		
Yes	1,522	81.9
No	337	18.1
Sought psych. Support because of the event		
Yes	135	7.1
No	1747	91.7
Prefer not to answer	22	1.2
If not, should you have?		
Yes	126	6.8
No	1,319	71.3
Not relevant	404	21.8
Medicated (anxiety, depression, sleeping problems) because of the event		
Yes	90	4.8
No	1778	94.5
Prefer not to answer	13	0.7
No		
On sick leave because of deployment		
Yes	55	3
No	1787	95.9
Not relevant	22	1.2

Nearly one-quarter reported having experienced one or more highly stressful events during their upbringing. Approximately 3.5% (*n* = 69) have been diagnosed with PTSD, 95.5% (*n* = 1805) have not been diagnosed with PTSD, and 0.5% (*n* = 12) preferred not to answer. For those who reported a diagnosis of PTSD, most were diagnosed after their reported deployment.

### Dropout analysis

The respondents who answered the questionnaire were compared to those who refrained from answering in terms of gender. Of those answering the questionnaire, 91.2% were men and 8.8% were women. The equivalent numbers among those who did not complete the questionnaire were 91.0% men and 9.0% women. The difference was statistically significant [chi-square (1) = 775, *p* = 0.001].

### Measures

#### Trauma exposure and PTSD indication

According to *the Diagnostic and Statistical Manual of Mental Disorders* (fifth edition; DSM–5), PTSD requires exposure to a traumatic or highly stressful event as a diagnostic criterion. Experience of a life-threatening traumatic event was studied using Criterion A (PCL-5) (Weathers et al., 1993). The scale was translated from English to Swedish by Nenad Paunović, a licensed psychotherapist and associate professor in psychology in 2015. The PTSD checklist (PCL-5, Weathers et al., 1993; Blevins et al., 2015) was used as an indicator of PTSD prevalence. The questionnaire consists of 20 questions with 5-point response scales, ranging from 0 (*Not at all*) to 4 (*Extremely*). The total score can range from 0 to 80, and the Cronbach’s alpha coefficient obtained was 0.94. Participants were asked to rate how much they had been affected by symptoms during the last month. According to the U.S. National Center for PTSD, previous research suggests that a PCL-5 cutoff score between 31 and 33 is indicative of probable PTSD across samples. However, the proposed cutoff scores should be interpreted with caution as they are preliminary, and additional research is needed because the population and the purpose of the screening might justify using different cutoff scores. Based on the previous preliminary findings, this study used a mean value (cutoff score) of 32.

#### Potentially morally injurious events (PMIEs) and moral injury symptoms

Exposure to potentially morally injurious events and the presence and severity of moral injury symptoms in the past month were measured using the Moral Injury Outcome Scale (MIOS-F), ranging from 0 (*Strongly disagree*) to 4 (*Strongly agree*), consisting of 14 items on a 5-point response scale (*α* = 0.90), measuring two symptom clusters: loss of trust and shame (Litz et al., 2021, 2022). The total score range is 0–56 for the overall scale and 0–28 for each of the subscales. Higher scores reflect increased levels of moral injury in the last month. There are currently no proposed scoring categories or cutoffs. In the Australian sample used for the MIOS validation study, a score of 37 or above in the MIOS was associated with functional impairment and was considered to indicate probable moral injury in this sample. As with PTSD cutoff scores, this score may differ for different study populations and should thus be interpreted with caution. Three categories are offered as a guide to assess symptom severity: 14–28 = *Mild*, 29–42 = *Moderate*, and 43–56 = *Severe*. However, these findings should also be interpreted with caution, and additional research is needed to determine the optimal severity score across different populations.

#### Combat exposure

The respondents’ overall combat exposure was investigated through the Combat Exposure Scale (Keane et al., 1989). Answers (raw scores) on the Combat Exposure Scale range from 1 to 5. Scores were converted to obtain a total score. The total exposure to combat was categorized according to 0–8 = *Light*, 9–16 = *Light – Moderate*, 17–24 = *Moderate*, 25–32 = *Moderate – Heavy*, and 33–41 = *Heavy*.

#### Milder symptoms

The Stress Profile Checklist was used to screen for milder symptoms (Setterlind and Larsson, 1995); this Checklist includes 12 psychological symptoms (emotional and cognitive). The study participants were asked to rate how often they had experienced the symptoms in the last month on a 5-point scale (1 = *Never*, 2 = *Seldom*, 3 = *Sometimes*, 4 = *Often*, 5 = *Very often*).

#### Homecoming and follow-up

The experience of homecoming and follow-up was studied through 10 self-constructed questions concerning reception and care, support and recovery, seeking support for mental problems, being on sick leave, perception of overall service, medication for anxiety, depression, or sleep difficulties due to the mission, and whether they had been diagnosed with PTSD. In some cases, respondents had the opportunity to provide additional comments on their answers.

#### Emotional stability

The Single Item Measure of Personality (SIMP; Woods and Hampson, 2005) was used to collect data on Emotional Stability (Costa and McCrae, 1992). The item is assessed on a 9-point rating scale. Despite the brief format and dichotomous constructs, the SIMP has been shown to have both convergent and divergent validity (Woods and Hampson, 2005).

### Statistical analysis

SPSS Statistics version 29 was used in the statistical analyses. Summary indices were calculated for all scales except the personality scales. Descriptive statistics were calculated. The data were analyzed using correlation and regression analyses to explore potential statistical relationships between variables of interest. Hierarchical regression analysis was performed with the PTSD indication scale as a dependent variable. Age, emotional stability, previous trauma, and alcohol problems were regarded as individual-related antecedent variables that were entered in Step 1. The two MIOS symptom-clusters, loss of trust and shame, were entered in Step 2. Data were analyzed using between-group analyses (*t*-tests and one-way between-group ANOVA with *post hoc* test) to examine significant differences between different groups.

#### Ethical considerations

When asked to participate, the potential respondents were provided with an information letter describing the purpose of the study, how the study would be conducted, potential risks following participation, and how the data would be used, stored, and protected by the project team. They were also asked to give their written informed consent to participate.

Although the study targeted voluntary participants, the content of the questionnaire could still be perceived as sensitive given, for example, the discussion of difficult experiences related to overseas service. Participants were informed that they could choose not to answer questions they considered too personal or sensitive. Additionally, participants were given a letter from the Swedish Armed Forces Veterans Center, which provided detailed information about where and to whom they could turn if they believed they needed support.

The entire research process was conducted in accordance with good research practice (Swedish Research Council, 2024) to minimize risks to research subjects, including respondent selection, data collection, data management, documentation, and data retention and storage. The project has received ethical approval from the Swedish Ethical Review Authority (Dnr 2023–04097-01).

## Results

Means, standard deviations, and bivariate correlations are presented in Table 3.

**Table 3:** Means, standard deviations and bivariate correlations.

Scale	*M*	*SD*	Phy.sym.	Emo.sym.	Cog.sym	PTSD.ind.	MIOS	Neg.alco.habits
Age	51.47	11.78	−0.001	−0.02	−0.01	−0.01	0.04	−0.01
Phy.sym.^a^	1.61	0.56		67^***^	62^***^	47^***^	0.45^***^	0.15
Emo.sym.^a^	1.64	0.71			0.78^***^	0.48^***^	0.58^***^	0.23^***^
Cog.sym. ^a^	1.70	0.79				0.51^***^	0.47^***^	0.19^***^
PTSD ind.^b^	4.31	8.40					0.71^***^	0.28^***^
MIOS^c^	7.15	8.29						0.25^***^
Neg. alco.habits^a^	1.51	0.66						

***p < 0.000 (2-tailed).

^a^Scores could range from 1 to 5.

^b^Scores could range from 0 to 85.

^c^Scores could range from 0 to 56.

As can be observed in Table 3, the mean scores on the symptom scales, the PTSD indication scale, and the MIOS scale are low. The milder symptoms scales, PTSD indication, and the MIOS were significantly and positively correlated with each other. Negative alcohol habits showed a small positive correlation with all mental symptoms’ scales.

### Prevalence of PTSD indication

Exposure to a traumatic or stressful event is a criterion for PTSD indication. Among the 1,940 individuals who completed the questionnaire, 62% (*n* = 1,121) gave accounts of a stressful event according to Criterion A that, more specifically, involved death or the threat of death, serious injury, or violence/sexual violence. Respondents described their experiences related to international deployment in the following ways: “The Srebrenica massacre,” “having been subjected to indirect fire at the camp,” “fired upon during a meeting with the local population,” “having been severely injured by a landmine in […], everyone else in the platoon died. They were from [name of countries],” “having been in continuous combat for 4 days,” “landmines on the roads we used. A local drove over a landmine with his family,” “I found myself between warring parties,” and “We had several suicides during my deployment.”

The data analysis showed that 96.4% (*n* = 1,080) of those fulfilling Criterion A did not show any prevalence of PTSD indication, whereas 3.6% (*n* = 40) did show the prevalence of PTSD.

### Prevalence of moral injury indication

To assess the prevalence of moral injury symptoms, such symptoms must be related to the experience of a potentially morally injurious event (PMIE). In the veteran sample, 26% (*n* = 504) of study participants reported having experienced a morally challenging situation during deployment. Respondents gave an account of such events in terms of “Same event as described earlier [trauma exposure, A criterion]. It should have been me who was affected…,” “Invasion in [name of country]. Our ambulances were not allowed to enter the city [name of city] that was bombed,” “Saw other countries’ soldiers bringing little children back to their rooms. Went around holding their hand and it was more of a sexual character than like a parent holding their child’s hand,” “We could offer very good care to [our men], but not to civilians. Like when we tried to get a civilian woman by helicopter to our [name of own camp] hospital, we were not allowed. They refused, of course. It was frustrating. She was the one most wounded during the mission,” “When a police post in the area nearby was attacked by [nationality], we prepared to rush to their rescue, but because of doubt and fear among our commanders and HQ, we were not allowed to go and rescue them, but they were left to their fate,” and “Srebrenica…powerlessness and frustration.”

Based on the cutoff score of 37, the prevalence of moral injury among the study sample was 1.6% (*n* = 8). Symptom severity among the respondents who experienced a PMIE is shown in Table 4.

**Table 4:** MIOS symptom severity (*N* = 545).

MIOS symptom severity (range 0–56)	% (*n*)
No symptoms (0–13 scores)	80 (435)
Mild (14–28 scores)	16 (88)
Moderate (29–42 scores)	3.7 (20)
Severe (43–56 scores)	0.4 (2)

Approximately 20% of the respondents indicated moral injury symptoms. A majority reported mild symptoms and only a few moderate or severe symptoms in the last month.

The study participants described their own role in the morally challenging event: (1) 39% (*n* = 198) referred to an event where they acted or failed to act; (2) 65.5% (*n* = 339) described having observed someone else’s actions (or their failure to act); and (3) 59% (*n* = 305) described being directly impacted by someone else’s actions (or their failure to act). However, as the survey questions describing the event were not mutually exclusive, respondents could answer yes to several of these questions. The nature of PMIE characteristics overlap is illustrated in Table 5.

**Table 5:** The characteristics of the potentially morally injurious events (PMIEs).

Characteristics of the event	% (*n*)	PTSD indication	MI indication
A. Did or failed to do something	11.9 (62)	8.1%	–
B. Observed someone else acting or failing to act	18.1 (93)	3.2%	–
C. Was directly impacted by someone else acting (or failing to act)	10.7 (54)	1.9%	1.9%
A + B	5.4 (28)	11.1%	7.1%
A + C	6.5 (33)	6.1%	-
B + C	27.6 (139)	7.2%	1.4%
A + B + C	14.4 (72)	12.3%	4.2%

Table 5 shows that most respondents gave an account of an event where they observed someone else’s actions or failure to act, while at the same time being directly impacted by someone else’s actions (or failure to act). Second, study participants described having observed someone else’s actions or failure to act. The third most common PMIE appears to be when all three dimensions coincide in the same event. According to Table 5, the highest prevalence of PTSD and MI indication is primarily among individuals who experienced PMIEs where all three dimensions coincide and, second, among individuals who felt they had acted while simultaneously observing another’s actions or failure to act, which contradicted deeply held values.

Tables 6, 7 give an overview of health-related outcomes in terms of PTSD and MI indication as related to the time passed since the stressful experience and gender.

**Table 6:** Time passed since the event – PTSD and MI indication.

Years passed since the event	Health-related outcome	
	PTSD indication^a^ *M*(SD)	MI indication^b^ *M*(SD)
0–10	6.30(10.5)	8.46(9.32)
11–20	6.03(10.31)	8.38(9.79)
21–32	4.80(8.29)	7.18(8.16)
> 32	3.09(9.36)	6.13(4.67)

^a^Scores could range from 0 to 80.

^b^Scores could range from 0 to 56.

**Table 7:** Gender – PTSD and MI indication.

Gender	Health-related outcome	
	PTSD indication^a^ *M*(SD)	MI indication^b^ *M*(SD)
Men	5.54(9.54)	7.97(9.09)
Female	4.85(9.70)	7.18(8.68)

^a^Scores could range from 0 to 80.

^b^Scores could range from 0 to 56.

There are no significant between-group differences in PTSD or MI indication scores regarding the time passed since the event.

There are no significant between-group differences in PTSD or MI indication scores regarding gender since the event.

The study participants’ overall combat exposure was also investigated and related to health effects last month. The mean value was 10.89 (*SD* = 7.50), which means that they rate their combat exposure at the lower end, between light and moderate. Table 8 gives a more detailed overview of the respondents’ overall combat exposure.

**Table 8:** Overall exposure to combat – PTSD and MI indication.

Exposure to combat (range 0–41)	%(*n*)	PTSD indication score^a^ *M(SD)*	MI indication score^b^ *M(SD)*
Light (0–8 scores)	60(1163)	3.37(6.97)	6.72(7.74)
Light – moderate (9–16 scores)	28(547)	5.89(9.29)	7.97(9.13)
Moderate (17–24 scores)	9.5(180)	8.98(12.49)	9.56(10.88)
Moderate – heavy (25–32 scores)	2(38)	9.71(13.20)	9.68(9.90)
Heavy (33–41 scores)	0.5(3)	38.67(25.70)	17.00(14.53)

^a^Scores could range from 0 to 80.

^b^Scores could range from 0 to 56.

Analysis of variance showed that there was a statistically significant overall difference at the *p* = <0.001 level in PTSD indication scores for all groups: *F*(4, 1,113) = 24.32. *Post hoc* comparisons showed significant (*p* < 0.05) pairwise differences for all comparisons except for those scoring moderate – heavy compared to light – moderate and moderate. There was no statistically significant difference between the groups regarding MI indication scores.

### Associations between PTSD and moral injury

Data analyses show that the strength of the correlation between the PTSD and MIOS scales is large (0.71, *p* < 0.001). There are significant associations between all symptom sub-clusters.

#### Type of stress exposure

According to the data analysis, groups of individuals describe exposure to different kinds of stress. Some respondents experienced an event according to Criterion A or a potentially morally injurious event, whereas other respondents gave accounts of exposure to both types of stress on different occasions. In other cases, exposure to both kinds of stress coincided with the same event. Table 9 gives an overview of the different types of stress exposure experienced by the study participants and their health-related outcomes in terms of the prevalence of PTSD and MI indication related to respective sub-groups.

**Table 9:** Overview of different kinds of stress exposure and health-related outcomes.

Trauma according to criterion A	Potentially Morally Injurious Event (PMIE)		
	Yes		No
Yes	Different occasions *n* = 288	The same event *n* = 78	*n* = 736
	PTSD indication: 5.2% (*n* = 15) MI indication: 1% (*n* = 3)	PTSD indication: 18.2% (*n* = 14) MI indication: 5.2% (*n* = 4)	PTSD indication: 1.4% (*n* = 10) No MI indication
No	*n* = 125 No MI indication		*n* = 562 No indication

According to Table 9, respondents who reported experiencing both types of trauma exhibited the highest prevalence of both PTSD and MI. For those individuals in whom both types of trauma coincided in the same event, the occurrence of both health outcomes increased significantly.

The relationship between the moral injury symptoms sub-cluster variables, loss of trust and shame, and the PTSD indication scale were also analyzed using multiple regression analysis. To account for the potential influence of common variance, the personality dimension, emotional stability, and experience of previous traumatic events were used. Additionally, as the symptoms must be unrelated to medication, substance use, other illnesses, or alcohol drinking habits (American Psychiatric Association, 2013, 2022), a scale measuring drinking habits was entered into the regression analysis.

Collinearity tests were performed (variation inflation factor – VIF) on the scales. The average VIF value on all scales was above 1, which is an acceptable result (Field, 2013). The results from the analysis with the PTSD indication scale as a dependent variable are shown in Table 10.

**Table 10:** Regression analysis (final model) – predictors on a PTSD indicator scale (ratings related to last month; *N* = 1,120).

Dependent variable and predictors	*b*	*SE B*	*B*	*F*	*p*
Individual antecedents^a^					
Age	−0.002	0.002	−0.037	−1.23	0.219
Emotional stability	−0.377	0.169	−0.072	−2.22	0.027*
Previous trauma	−2.50	0.667	−0.114	−3.74	0.001**
Combat exposure	0.219	0.044	0.152	4.99	0.001**
Alcohol problems	0.248	0.435	0.018	0.57	0.569
Moral injury-related symptoms last month^b^					
MIOS Loss of trust	0.291	0.068	0.171	4.28	0.001**
MIOS Shame	1.429	0.102	0.598	13.96	0.001**

^a^Individual antecedents; age, emotional stability, previous trauma, combat exposure, alcohol problems, were entered in Step 1, ^b^Moral injury-related symptoms; MIOS Loss of trust and MIOS shame, were entered in Step 2. Step 1: *R*^2^ = 0.25, adjusted *R*^2^ = 0.24; Step 2: *R*^2^ = 0.66, adjusted *R*^2^ = 0.65. *R*^2^ change between Step 1 and Step 2 is significant (*p* < 0.001). * *p* < 0.05, ** *p* < 0.001.

Table 10 shows that a high adjusted R^2^ value was obtained on the PTSD indication scale, showing that emotional stability, previous trauma, combat exposure, and both MIOS symptom clusters, particularly shame, showed a significant association with the dependent variable.

#### Subgroup comparisons

Among the study participants who reported having experienced a morally challenging event (PMIE), subgroup comparisons were made between respondents who experienced *both* trauma types (1) on different occasions and (2) those who gave accounts of the two trauma types coinciding in the same event.

The respondents who experienced both trauma types (not necessarily in one event) scored significantly higher on the PTSD and moral indication scales and on the emotional and cognitive symptoms scales, higher on openness, and lower on the personality dimension emotional stability.

Statistical subgroup comparisons were also made among respondents who had experienced both trauma types, based on the subgroups presented in Table 1 (general background questions, such as gender, age, education, and branch of service) and Table 2 (health- and deployment-related questions). The results are presented in Table 11.

**Table 11:** Subgroup comparisons based on general background and health- and deployment-related questions.

PTSD indicator scale (range 0–80)	Yes			No			*t*(df)	*p*	Cohen’s *d*
	*n*	*M*	*SD*	*n*	*M*	*SD*			
Previous trauma experience	135	10.81	14.34	231	6.96	10.86	2.70(224.307)	0.01	6.66
Previously diagnosed with PTSD^a^	33	27.27	3.34	327	6.45	9.80	6.16(33.709)	0.001	1.90
Did not receive sufficient support or recovery post-deployment	249	5.44	8.22	112	15.52	17.20	−5.90(134.324)	0.001	−0.86
Sought support for psychological distress caused by the event^a^	58	22.64	18.93	301	5.37	8.02	6.83(60.995)	0.001	1.64
Did not seek support for psychological distress related to the event, but believe they should have^b^	51	15.35	16.03	234	4.34	5.73	4.84(52.813)	0.001	1.29
Used medication for anxiety, depression and sleeping difficulties due to the deployment^a^	45	27.53	18.87	314	5.55	8.09	7.72(46.344)	0.001	2.19
Needed sick leave due to mental health issues after deployment^a^	21	30.14	19.84	335	6.70	6.70	5.37(20.645)	0.001	2.17
Perceived that reception or care upon returning home was inadequate or poor	151	13.19	16.22	214	5.35	7.80	−5.51(199.195)	0.001	−0.65
Considered leaving the Armed Forces but did not, and those who left their employment	82	15.55	16.42	274	6.25	10.08	−4.86(99.934)	0.001	−0.79

^a^Those who indicated “Prefer not to answer” are not included in the analyses.

^b^Those who indicated “Not applicable” are not included in the analyses.

## Discussion

The first aim of the study was to explore the prevalence of indications of PTSD and moral injury in Swedish military veterans. In the study sample of 1,940 veterans, 1,121 (62%) met the PTSD Criterion A. Based on the PTSD indicator scale (Weathers et al., 1993), 3.6% (*n* = 40) scored above the cutoff score of 32. This score is a low proportion, and this result is comparable to earlier Swedish studies exploring health among military veterans at the group level (Larsson et al., 2017; Neovius Pousette et al., 2023a). We suggest that a major reason for this finding is the Swedish Armed Forces’ selection requirements, which ensure that those eligible for international military missions are physically and mentally healthier than the general population (McLaughlin and Waller, 2008). Another reason could be the so-called dose–response relationship, i.e., the study group’s relatively limited overall experience of highly stressful situations (McLean et al., 2013).

Turning to moral injury, 504 (26%) respondents reported that they had experienced a morally challenging situation. Using the MIOS scale and a cutoff score of 37, 1.6% (*n* = 8) met the criterion of moral injury indication, an even lower proportion than that found in the assessment of PTSD indication. There is no previous Swedish military reference data available; nevertheless, the finding of 1.5% is low. Our best guess is regarding resilience factors, which are related once again to the stringent selection requirements and the stress dose–response of the Swedish Armed Forces investigated in this study.

To summarize, the vast majority of the military veterans studied did not exhibit risk scores related to PTSD or moral injury. However, apart from the small group that met the cutoff score criteria, several respondents reported milder symptoms of psychological and/or moral distress.

The second aim concerned the relationship between PTSD and moral injury indications. The overall bivariate correlation between the two scales was 0.71 (*p* < 0.000), which indicates a high degree of interrelatedness. Similar results were found when the subscales of the two instruments were analyzed. Correlations ranging from 0.58 to 0.81 were found. Thus, these findings suggest that there is a considerable overlap between the two theoretical constructs. However, it has been suggested that one challenge in interpreting associations between PTSD and moral injury is the possibility of overlapping trauma types, e.g., if an index event, to which an individual was exposed, was both potentially life-threatening and a morally injurious event (Stein et al., 2012).

An alternative approach to exploring the relationship between PTSD and moral injury indications is to compare respondents with different kinds of stressful exposures. Among the 1,121 respondents who experienced an event in accordance with Criterion A and the 504 who experienced a morally challenging event, 288 reported two different events and 78 reported the same situation. There was a considerably higher prevalence of PTSD (18.2%) and MI indication (5.2%) in the group that reported the same event. The MI symptom subcluster shame accounted for the largest variance in the PTSD indicator scale within the study sample.

The results show that the risk of developing PTSD and MI is highest when an event is simultaneously perceived as very stressful (fear-based) and morally challenging. This suggests that PMIEs tend to have worse effects (more severe and prolonged stress reactions) when they coincide with acute traumatic events. One explanation may be that, during traumatic situations (according to Criterion A), individuals often experience high levels of stress, which can lead to a relapse into instinctive behavior and impaired cognitive function (e.g., Janis, 1986). Jones (2006) describes how people who fear for their lives neither have the time nor the inclination to register their own thoughts and feelings, which may undermine their ability for moral reflection. For this reason, there may be a greater tendency to question one’s own moral choices and blame oneself after the event for less successful decisions made during the acute phase, once they have regained full cognitive functioning. Further studies are obviously needed to substantiate this hypothesis. Another valuable area for further research could involve a closer examination of the relationship between moral injury and complex PTSD, a form of PTSD that encompasses disturbances in the concept of self and interpersonal relationships, where self-blame is commonly observed (e.g., Currier et al., 2021).

The third and last aim of the study was to explore how PTSD and moral injury indications are related to a selection of background variables. Beginning with PTSD indication, a multiple regression analysis showed that emotional stability, one or more severe traumas during childhood, and combat exposure contribute positively to PTSD indication. This finding is well documented in previous research (e.g., Fear et al., 2010; Syed Sheriff et al., 2020).

Furthermore, those who scored higher on the PTSD indicator scale in the past month reported - to a greater extent - a lack of support and recovery, seeking psychological help, being on sick leave, and the use of medication due to stressful experiences after deployment. Additionally, they expressed greater dissatisfaction with the deployment. These factors may serve as early indicators of long-term ill health shortly after returning home.

The higher prevalence of indication of PTSD and MI in the past month, despite receiving various forms of support upon their return, suggests that individuals still attribute their distress to their participation in military deployment. Van der Wal et al. (2020) recently conducted a study on PTSD symptoms among military veterans and identified a group of individuals who, like the participants in this study, did not appear to improve despite receiving mental health treatment. They conclude that pre-existing vulnerabilities in these individuals remain relevant even 10 years later and argue for alternative treatment methods. In alignment with this conclusion, this study points to the importance of incorporating moral injury treatment as a complement to more established clinical approaches for fear-based trauma exposure and PTSD symptoms, as suggested by Steenkamp et al. (2015).

Finally, there are some methodological considerations. Drawbacks include the cross-sectional study design, self-report data of symptoms of PTSD instead of formal PTSD diagnostics, and the comparatively low response rate, particularly the proportion of female respondents. This last aspect made detailed gender comparisons impossible in terms of the analyses of subgroups with high PTSD and moral injury indication scores. It is possible that a similar study involving only women military veterans could yield a higher response rate. This is suggested as an area for further research. Other limitations and areas for future studies include gathering more data on military organization, leadership, peer support, and other service conditions. A key strength of the study is its large sample size and the use of well-established measurement scales.

### Main findings and clinical implications for practitioners

The risk of ill health, in the form of PTSD and MI, appears to be highest when an event is simultaneously perceived as both very stressful (fear-based) and morally challenging (particularly shame-based within the study sample). This alternative approach to exploring the relationship between PTSD and moral injury indications, by comparing respondents with different types of stressful exposures, proved fruitful and adds to the current understanding of MI.

Health- and deployment-related background variables that may be associated with indications of PTSD and moral injury were identified. Regarding practical implications, the study results highlight the types of stressful experiences, as well as health- and deployment-related factors, that should be monitored post-deployment. These factors may serve as significant risk indicators for the development of PTSD and moral injury.

The higher prevalence of PTSD and MI indications in the past month, despite receiving various forms of support upon returning from deployment, highlights the need for MI-specific treatment.

## Data Availability

The raw data supporting the conclusions of this article may be requested from the authors.
